# Expected small left heart size in the presence of congenital diaphragmatic hernia: Fetal values and Z-scores for infants confirmed to have no heart disease postnatally

**DOI:** 10.3389/fped.2022.1083370

**Published:** 2022-12-06

**Authors:** Anita J. Moon-Grady, Francesca A. Byrne, Leslie A. Lusk, Roberta L. Keller

**Affiliations:** ^1^ Division of Pediatric Cardiology, University of California San Francisco, Benioff Children’s Hospital, San Francisco, CA, United States; ^2^ Fetal Treatment Center, University of California San Francisco, Benioff Children’s Hospital, San Francisco, CA, United States; ^3^ Pacific Cardiovascular Associates, Pediatric Cardiology, Orange, CA, United States; ^4^ Division of Neonatology, Department of Pediatrics UCSF Benioff Children's Hospital, Oakland, CA, United States; ^5^ Division of Neonatology, University of California San Francisco, Benioff Children’s Hospital, San Francisco CA, United States

**Keywords:** fetal echocardiography, lung hypoplasia, pulmonary hypertension, vascular hypoplasia, ventricular hypoplasia

## Abstract

**Objectives:**

In fetuses with left-sided congenital diaphragmatic hernia (CDH), left heart structures may appear small, but usually normalize after birth in the absence of structural cardiac anomalies. To decrease the possibility of an erroneous diagnosis of structural heart disease, we identify normal values for left heart structures in the presence of left CDH and secondarily investigate the relationship of left heart size and survival to neonatal hospital discharge.

**Methods:**

Left heart structures [mitral valve (MV) and aortic valve (AoV) annulus diameter, left ventricle (LV) length and width] were measured by fetal echocardiogram in fetuses with left CDH and no congenital heart disease. We generated linear regression models to establish the relationship of gestational age for each left heart structure using data from fetuses who survived after birth. We calculated z-scores (normalized to gestational age), and assessed the relationship of survival to the size of each structure.

**Results:**

One hundred forty-two fetuses underwent fetal echocardiogram (median 25 weeks' gestation, IQR 23, 27 weeks). Left heart structures were deemed small when using published normative data from unaffected fetuses (z-scores: MV −1.09 ± 1.35, AoV −2.12 ± 1.16, LV length −1.36 ± 1.24, LV width −4.79 ± 0.79). CDH-specific models derived from log-transformed values yielded left-shifted distributions, reflecting the small structures (mean z-score lower by: MV 0.99 ± 0.30, AoV 2.04 ± 0.38, LV length 1.30 ± 0.36, and LV width 4.69 ± 0.28; *p* < 0.0001 for all comparisons). Non-survivors had worse z-scores than survivors for all measurements, but this did not reach statistical significance.

**Conclusions:**

Log-transformed linear models generated new normative data for fetal left heart structures in left CDH, which may be used to allay antenatal concerns regarding structural left heart anomalies. There were no significant differences in z-scores between survivors and non-survivors, suggesting that in the absence of true structural disease, cardiac evaluation is not predictive in isolation and that causes of mortality are likely multifactorial in this population.

## Introduction

Congenital diaphragmatic hernia (CDH) is characterized by bilateral lung parenchymal and vascular hypoplasia (1–9). Associated anomalies may occur, including congenital heart disease, which carries a worsened prognosis (10–14). However, independent of structural cardiac defects, prior work described small left-sided heart structures after birth, particularly in infants with circulatory derangements associated with persistent pulmonary hypertension of the newborn (PPHN) (15–17). This physiology results in blood shunting away from the pulmonary circulation with decreased left heart filling and output, making definitive conclusions regarding the underdevelopment of structures difficult. Additional studies have described small fetal left-sided structures, compared to gestational age (GA)-specific norms from unaffected fetuses, although the size of these structures significantly improves after neonatal repair of the diaphragmatic defect (5, 16, 18–25).

Consistent with variation in the size of fetal left heart structures, we and others have described differences in fetal blood flow, as indicated by output measurements from the right ventricle (RV) and left ventricle (LV), measured flows in the pulmonary circulation and the ductus arteriosus (DA), and the path of umbilical venous flow into the heart (22–25). LV output is shown to be decreased in fetuses with both left and right CDH, and fetuses with markers of more severe left CDH have more profound discrepancies than less seriously affected fetuses with left CDH and those with right CDH. These alterations are associated with increased DA blood flow (pulmonary artery-to-aorta), decreased pulmonary blood flow, and variably decreased flow (right-to-left atrium) *via* the foramen ovale (24, 25).

Thus as the left heart is expected to be small in the fetus with CDH, it is difficult to interpret fetal heart measurements obtained using published normative values derived from fetuses without CDH. Standardized values for left heart structures in fetuses with CDH but without congenital heart disease have not been defined. We first aimed to define the normal size of left-sided heart structures in fetuses with left CDH without significant structural cardiac disease who survived postnatally and were confirmed to have no heart disease. We hypothesized that normal CDH-specific curves would be left-shifted compared to curves generated from normal fetuses. Secondarily, we investigated whether the size of left-sided heart structures [mitral valve (MV) and aortic valve (AoV) annulus diameters and LV length and width] normalized for gestational age using CDH-specific data, was associated with neonatal survival in fetuses without congenital heart disease.

## Materials and methods

We conducted a retrospective study of fetuses evaluated by the University of California San Francisco (UCSF) Fetal Treatment Center with diagnosis of CDH (2000–2010). All fetuses underwent ultrasound evaluation at UCSF, with anatomic description of the CDH [including lung-to-head ratio (LHR) (26)], and documentation of other associated anomalies. Fetal echocardiography was performed by sonographers with expertise in fetal echocardiography under the supervision of pediatric echocardiographers with expertise in fetal diagnosis using ultrasonography systems (Siemens S2000 or Acuson Sequoia C256 and C512; Siemens Corp., Mountain View CA, USA) equipped with a combination of curvilinear and phased array probes operating at 6–8 MHz. All studies included a complete two-dimensional evaluation of cardiac structures and systolic ventricular function with complete pulsed-wave and color Doppler examinations including venous and umbilical cord investigations. Fetal echocardiograms were retrospectively reviewed and measurements made offline using a commercially available workstation (Syngo Dynamics, Siemens Healthcare GmbH) by a single pediatric cardiologist (FAB) blinded to clinical outcome, from digital images stored in standard DICOM format. Measurements were as previously described (25, 27) and included:

• The mitral valve and tricuspid valve (TV) annulus diameters in the 4-chamber view during the peak filling in early diastole at maximum excursion of the leaflets from inner edge to inner edge at hinge points of the leaflet attachments,
• The aortic valve and pulmonary valve (PV) annulus diameters in long axis views at the inner edge of the annular hinge points at maximal opening in systole,
• Left ventricle major axis length (LV length) measured at end-diastole, defined as the frame at which the MV and TV closes, taking care not to foreshorten the ventricles, from the middle of the annulus level to the apical endocardium, and
• Left ventricle short axis (LV width) dimension at end-diastole (maximal size) at the level of the papillary muscles

GA-specific z-scores were generated for each measurement based on prior studies of fetuses unaffected with CDH or congenital heart disease, with GA specified as completed weeks by best obstetrical dating. We used published equations for predicted left heart parameters from McElhinney and colleagues and we assessed the robustness of our findings and any influence on right heart structures with application of widely-used equations derived by Schneider et al. (28, 29). The study was conducted under UCSF Institutional Review Board approval with expedited review and waiver of consent.

Measurements from a single evaluation of consecutive fetuses were included if newborn was liveborn and cared for at UCSF Children's Hospital after birth, and fetal cardiac structural measurements were possible from the available stored echocardiogram images. Fetuses with abnormal karyotype, suspected or confirmed genetic syndromes, and those undergoing fetal tracheal occlusion were excluded (27). Neonatal inclusion criteria included confirmation of left CDH and exclusion of congenital heart disease (other than patent ductus arteriosus and patent foramen ovale), based on clinical examination and postnatal echocardiogram, and no need for cardiac surgery or catheter intervention. Data were maintained in a REDCap (Research Electronic Data Capture) database.

Statistical analyses were undertaken for each cardiac parameter (Stata version 11.2, College Station TX). Cardiac parameters were plotted vs. GA to assess for linearity with and without log transformation with a goal of identifying the best approach for all parameters; prior published work presents models with both non-transformed and transformed data (28, 29). Linear regression equations for each predicted parameter measurement were generated for two groups. First, we considered all fetuses without significant congenital heart disease that ultimately survived to hospital discharge. Residual plots confirmed the best fit models overall for the parameters (non-transformed vs. transformed models), and z-scores for the structures for fetuses with CDH were generated from these models. Regression equations were generated using all available data, with observations omitted only from equations specific to the absent measurement. Next, since mortality in newborns with CDH is not always directly related to cardiopulmonary development, we similarly evaluated models using data from all fetuses without significant congenital heart disease, regardless of survival. We assessed for an interaction between survival and gestational age at measurement, to identify if the change in these structures over gestation differed based on survival status. In the case of a significant interaction, stratified models generated from only survivors might better represent normal values. Then, the relationship of fetal z-scores to survival to discharge from the neonatal hospitalization was assessed for each left heart structure by *t*-test, using both published GA-specific norms for unaffected fetuses and the left CDH-specific norms generated from the current study. Finally, logistic regression was used to derive area under the receiver-operator characteristic (AUROC) curve with CDH-specific z-scores, to further assess the utility of fetal left heart structures for survival.

## Results

### Patient characteristics

Of referrals to the UCSF Fetal Treatment Center during the study period, 197 with documented left CDH without abnormal karyotype or genetic syndrome underwent imaging evaluation by ultrasound and echocardiogram (including measures of cardiac structure). Nine underwent fetal tracheal occlusion, 15 had a diagnosis of congenital heart disease, and 31 had incomplete follow up or inadequate images available for review, leaving 142 fetuses eligible for the study, with evaluation at 18–37 weeks' GA. At the time of the evaluation, 136 fetuses had LHR measured. Cardiac parameters obtained at fetal echocardiogram performed at median of 25 weeks' GA (range 18–37 weeks) are shown, with no missing data for MV and TV diameters, and limited missing data for other parameters (Table 1). There were no significant differences in LHR or survival after birth between those with (*n* = 22) and without one or more missing parameters (1.07 ± 0.32 and 1.27 ± 0.76, *p* = 0.22% and 59% vs. 66%, *p* = 0.54, respectively). The distribution of right-sided cardiac measurements was similar to that of unaffected fetuses. Left-sided cardiac measurements, however, were generally small, with LV width the most affected measurement. Differences in predicted parameters by the two sets of prediction equations were not substantial, with Schneider predictions slightly higher than McElhinney predictions for MV annulus diameter, and slightly lower for AoV annulus diameter (mean differences 0.08 ± 0 cm and −0.05 ± 0.02 cm for mitral and aortic valves, respectively). However, these differences translated into a more substantial, inverted, difference in z-scores, with the standardized Schneider scores lower than McElhinney scores for MV annulus diameter and higher than McElhinney scores for AoV annulus diameter (mean −0.82 ± 0.30 and 0.87 ± 0.27 for mitral and aortic valves, respectively).

**Table 1:** Fetal echocardiographic parameters from fetuses with left CDH with z-scores from published equations derived from unaffected fetuses.

Echocardiographic parameter	Measurement	z-score^a^ (McElhinney et al. 2009)	z-score^b^ (Schneider et al. 2005)
Gestational age at exam (weeks, *n* = 142)	25 (23, 27)		
Mitral valve diameter (cm, *n* = 142)	0.59 ± 0.14	−1.09 ± 1.35	−1.91 ± 1.27
Aortic valve diameter (cm, *n* = 133)	0.34 ± 0.07	−2.12 ± 1.16	−1.25 ± 1.20
Left ventricle length (cm, *n* = 140)	1.55 ± 0.36	−1.36 ± 1.24	
Left ventricle width (cm, *n* = 124)	0.34 ± 0.13	−4.79 ± 0.79	
Tricuspid valve diameter (cm, *n* = 142)	0.78 ± 0.19		−0.07 ± 1.24
Pulmonic valve diameter (cm, *n* = 136)	0.50 ± 0.11		0.35 ± 1.04

Data presented as median (interquartile range) or mean ± SD.

^a^ z-score determined by published equations from McElhinney et al. 2009 (29). Data presented as mean ± SD.

bz-score determined by published equations from Schneider et al. 2005 (28). Data presented as mean ± SD.

### Statistical analysis and generation of Z-scores

The best linear representation for the relationship of GA to the four left heart parameters was by log transformation, as applied in prior studies (28). Equations for all measured parameters are presented (Table 2). Derived predicted mean values and z-score for a given gestational age for each cardiac parameter are:

(1) Mean predicted value = exp [(*β* * gestational age) + constant]
(2) z-score = [(ln(measured value) − ln(predicted value)]/root MSE

**Table 2:** Equations for prediction of echocardiographic parameters in fetuses with left CDH that survived after birth.

Parameter	Coefficient (*β*)*	Constant	Root MSE	Adjusted *R*2
MV diameter (*n* = 92)	1.2883	−4.6913	0.1521	0.62
AV diameter (*n* = 87)	1.1508	−4.8060	0.1513	0.57
LV length (*n* = 90)	1.1145	−3.1666	0.1771	0.47
LV width (*n* = 81)	1.3237	−5.3737	0.3022	0.31
TV diameter (*n* = 92)	1.2398	−4.2647	0.1702	0.54
PV diameter (*n* = 89)	1.2327	−4.6852	0.1256	0.68

Mean predicted value = exp [(β * gestational age) + constant].

MV, mitral valve; AV, aortic valve; LV, left ventricle; TV, tricuspid valve; PV, pulmonic valve.

*All coefficients were statistically significant (*p* < 0.001).

**Figure 1:**
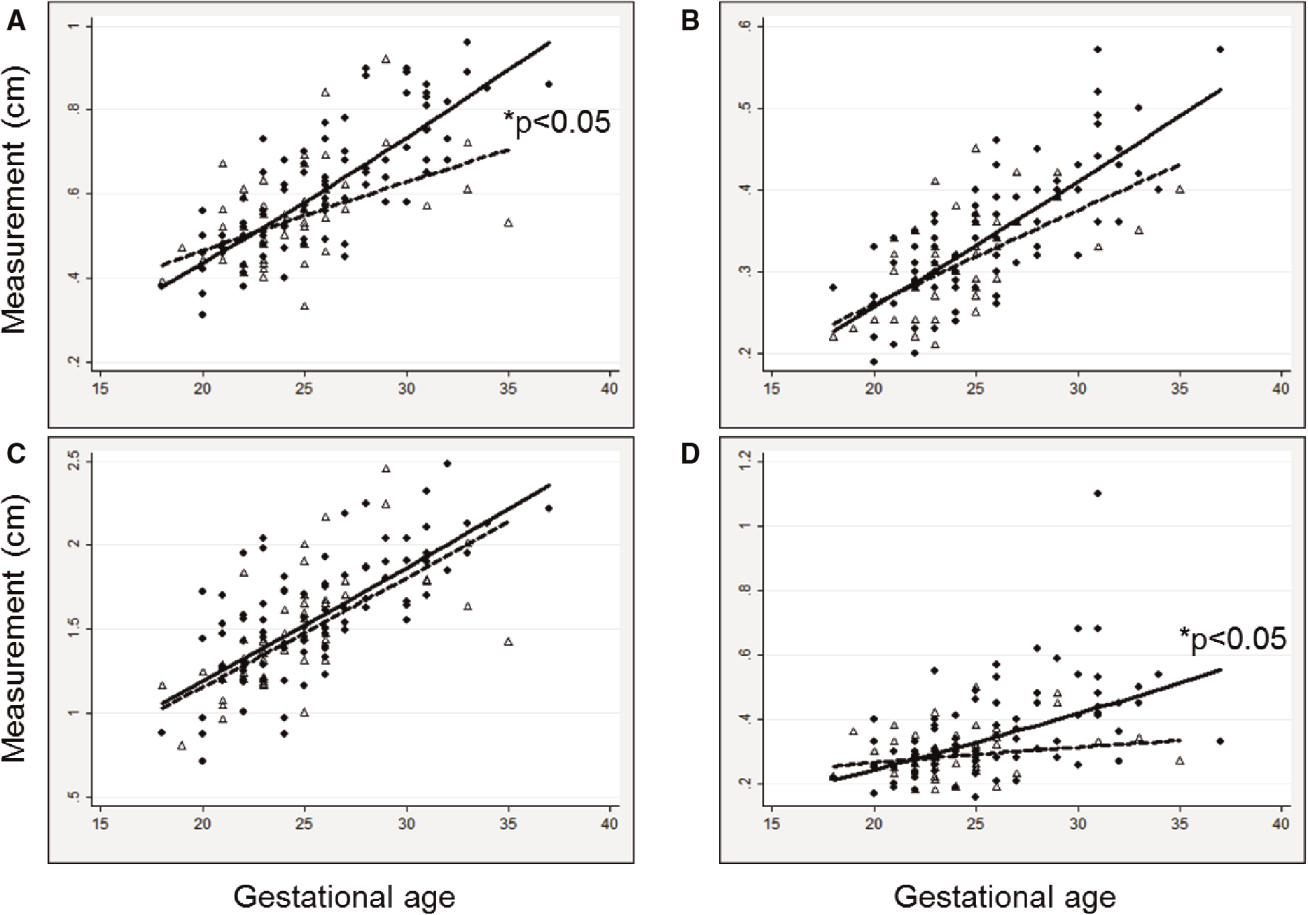
Measured left heart structures and left CDH-specific regression lines versus gestational age at measurement in survivors (solid circles, solid line) and a comparison group of non-survivors (open triangles, dashed line), (**A**) mitral valve diameter; (**B**) aortic valve diameter; (**C**) left ventricle length; (**D**) left ventricle width. Regression lines are back-transformed from natural log models. *P-value for interaction between survival and gestational age.

**Figure 2:**
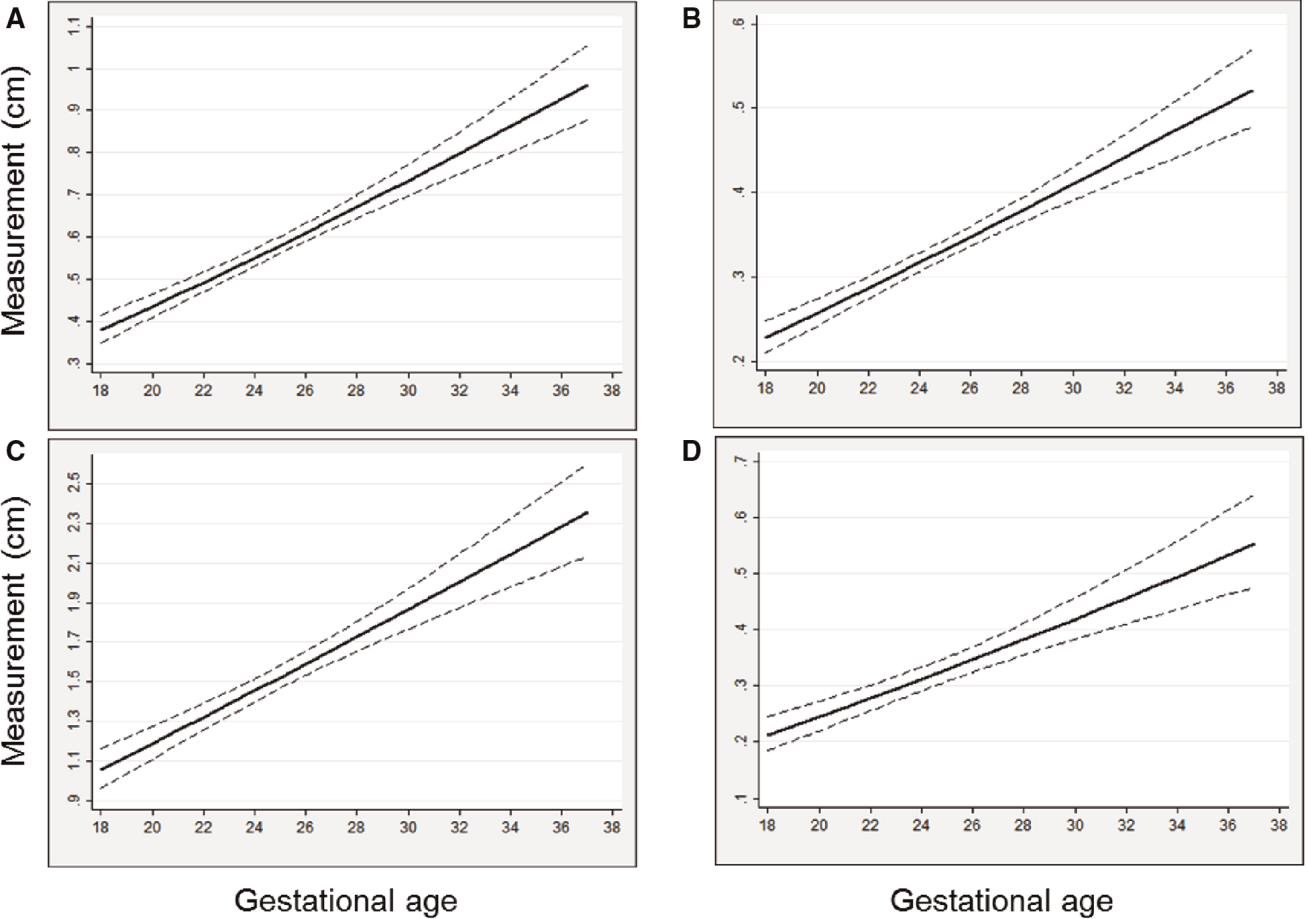
Predicted left heart structure size versus gestational age from left CDH-specific models, with regression line and 95% confidence interval shown, (**A**) mitral valve diameter; (**B**) aortic valve diameter; (**C**) left ventricle length; (**D**) left ventricle width. Predicted values are back-transformed from natural log models.

**Figure 3:**
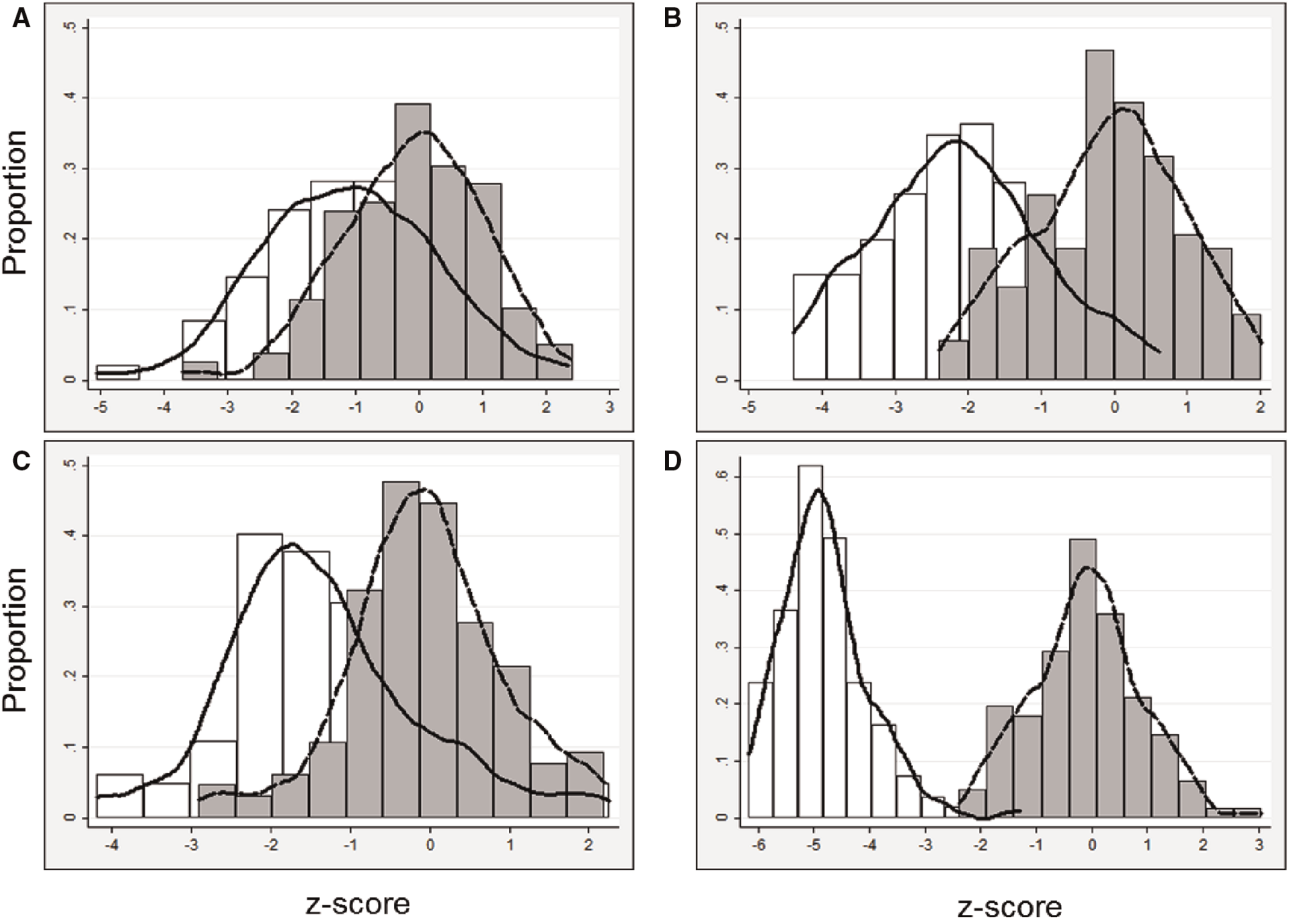
Left heart parameters (data derived from survivors only): distribution of z-scores and kernel density estimates from all fetuses with left CDH derived from (1) published data from unaffected fetuses (29) (white bars, solid line) and (2) left CDH-specific data (gray bars, dashed line). CDH-specific equations were derived with data only from survivors, (**A**) mitral valve diameter, *n* = 92; (**B**) aortic valve diameter, *n* = 87; (**C**) left ventricle length, *n* = 90; (**D**) left ventricle width, *n* = 81.

In general, models derived from data limited to fetuses with survival after birth (*n* = 92) better explained the relationship between gestational age and each measurement than models including all fetuses (*n* = 142, Supplementary Table). For each parameter, actual measurements are plotted vs. gestational age, with the overlying linear CDH-specific prediction for survivors and non-survivors (Figure 1). Interaction terms for survival and gestational age at time of measurement were significant for mitral valve annulus diameter (*p* = 0.009) and left ventricular width (*p* = 0.02) in models generated with data from both survivors and non-survivors, supporting the generation of normative values from models developed with data from only survivors (also shown with 95% confidence intervals, Figure 2). There were weak positive correlations between concurrent LHR and MV and AoV diameter (Table 3) though only LHR vs. AoV reached statistical significance. Right heart structures did not correlate with LHR measurements. The distributions of fetal z-scores for each parameter derived from unaffected fetuses and the left CDH-specific equations are also shown (Figure 3 for models generated from survivors only, and Supplementary Figure for models generated from both survivors and non-survivors). As expected from these graphs, CDH-specific z-scores generated with data from survivors only were significantly worse than z-scores from unaffected fetuses for all left heart parameters, with the largest differences for LV width, followed by AoV diameter: MV diameter 0.99 ± 0.30, AoV diameter 2.04 ± 0.38, LV length 1.30 ± 0.36, and LV width 4.69 ± 0.28; *p* < 0.0001 for all comparisons by paired t-test.

**Table 3:** The relationship of low lung-to-head ratio (≤ 1.0) and cardiac structure z-scores, based on CDH-specific prediction equations.

Parameter	LHR > 1.0	LHR ≤ 1.0^a^	*p*-value*
MV diameter	0.08 ± 1.10	−0.27 ± 1.11	0.07
AV diameter	0.16 ± 0.98	−0.27 ± 1.01	0.02
LV length	0.04 ± 1.09	−0.14 ± 0.90	0.31
LV width	0.06 ± 0.99	−0.27 ± 0.88	0.06
TV diameter	0.05 ± 0.99	−0.10 ± 1.00	0.37
PV diameter	−0.06 ± 1.16	0 ± 0.82	0.73

Data are presented as mean ± SD.

LHR, lung-to-head ratio; MV, mitral valve; AV, aortic valve; LV, left ventricle; TV, tricuspid valve; PV, pulmonic valve.

^a^ Overall, there were 71 fetuses with LHR ≤ 1.0 and 65 fetuses with LHR > 1.0. Data were complete for MV and TV diameter. For AV diameter, there were data on 67 with LHR ≤ 1.0 and 60 with LHR > 1.0, for LV length, there were data on 70 with LHR ≤ 1.0 and 64 with LHR > 1.0, for LV width there were data on 62 with LHR ≤ 1.0 and 56 with LHR > 1.0, and for PV diameter, 66 with LHR ≤ 1.0 and 64 with LHR > 1.0.

*p-value by *t*-test.

Though the focus was to generate normal values for left heart dimensions in the presence of CDH and clear absence of structural heart disease (hence limitation to a cohort who survived the neonatal period with normal postnatal cardiac examination for generation of equations) we secondarily evaluated the relationship of the size of fetal cardiac structures to survival to hospital discharge in our larger cohort. We used both z-scores from unaffected fetuses and the z-scores derived from our left CDH cohort for these analyses. Overall survival after birth was 65% (92/142). Although fetal z-scores from infants who died were lower than z-scores for those who survived for all parameters, the differences were not statistically significant, regardless of the equation used to derive z-scores (Table 4). Consistent with these findings, AUROC demonstrated that z-scores derived from the CDH equations had limited utility for the outcome of mortality: MV diameter 0.57 (95% CI, 0.46, 0.67), AoV diameter 0.57 (95% CI, 0.47, 0.68), LV length 0.55 (95% CI, 0.45, 0.65), LV width 0.58 (95% CI, 0.47, 0.69). We further evaluated performance of fetal left heart measurements for mortality with a threshold z-score <−1. This z-score corresponds to the 16th percentile for a normal distribution, it encompassed 14%–22% of observations from this cohort (depending on the individual measurement), and it included varying proportions of those observations with a z-score <−2 (<2.5% of normal values) when using data from unaffected controls: MV diameter, 31/37 (84%); AoV diameter, 28/66 (42%); LV length, 20/43 (47%); LV width, 23/123 (19%). CDH-specific left heart parameters performed modestly with a cut-off of z-score <−1; for all parameters, 61%–63% of newborns were classified correctly (Table 5). There was low sensitivity but good specificity, indicating that survivors were likely to have a z-score of −1 or higher. Positive and negative predictive values were poor and moderate, respectively.

**Table 4:** Survival to discharge and z-scores for cardiac structures derived from equations from CDH-specific and unaffected fetuses.

Parameter	CDH-specific		McElhinney et al.^a^		Schneider et al.^b^	
	Survivors	Non-survivors^c^	Survivors	Non-survivors	Survivors	Non-survivors
MV diameter	0 ± 0.99	−0.27 ± 1.28	−0.99 ± 1.22	−1.27 ± 1.55	−1.78 ± 1.16	−2.14 ± 1.44
	*p* = 0.16		*p* = 0.24		*p* = 0.11	
AV diameter	0 ± 0.99	−0.22 ± 1.03	−2.08 ± 1.13	−2.19 ± 1.21	−1.17 ± 1.18	−1.39 ± 1.24
	*p* = 0.23		*p* = 0.60		*p* = 0.33	
LV length	0 ± 0.99	−0.17 ± 0.96	−1.30 ± 1.28	−1.47 ± 1.17		
	*p* = 0.33		*p* = 0.44			
LV width	0 ± 0.99	−0.28 ± 0.91	−4.72 ± 0.83	−4.91 ± 0.72		
	*p* = 0.12		*p* = 0.20			
TV diameter	0 ± 0.99	−0.18 ± 1.00			−0.01 ± 1.23	0.18 ± 1.26
	*p* = 0.31				*p* = 0.44	
PV diameter	0 ± 0.99	−0.06 ± 0.98			0.35 ± 1.04	0.34 ± 1.04
	*p* = 0.75				*p* = 0.96	

Data presented as mean ± SD.

MV, mitral valve; AV, aortic valve; LV, left ventricle; TV, tricuspid valve; PV, pulmonic valve.

^a^ z-score determined by published equations from McElhinney et al. 2009 (29).

^b^ z-score determined by published equations from Schneider et al. 2005 (28).

^c^ Overall, there were 92 survivors and 50 non-survivors. Data were complete for MV and TV diameter. For AV diameter, there were data on 87 survivors and 46 non-survivors, for LV length, there were data on 90 survivors and 50 non-survivors, for LV width, there were data on 81 survivors and 43 non-survivors, and for PV diameter, 89 survivors and 47 non-survivors.

**Table 5:** Test diagnostics of CDH-specific fetal left heart measurement using a threshold of z-score <−1 for mortality prior to discharge from neonatal hospitalization.

Parameter	Sensitivity	Specificity	PPV	NPV	Correctly classified
MV diameter	26%	80%	42%	67%	61%
AV diameter	24%	80%	39%	67%	61%
LV length	16%	87%	40%	65%	61%
LV width	23%	84%	43%	67%	63%

PPV, positive predictive value; NPV, negative predictive value; MV mitral valve; AV aortic valve; LV left ventricle.

## Discussion

Fetal evaluation of CDH provides important information for families and clinicians as they consider fetal and neonatal interventions and outcomes. Fetuses with anomalies in addition to the CDH, and in particular cardiac anomalies, have a significantly worse prognosis. Many fetuses with CDH may appear to have small left heart structures, but though relative hypoplasia of the mitral and aortic valves and left ventricle raise the possibility of true structural heart disease in these fetuses, the size of most structures will in fact normalize after birth (23). We present normative values for left heart structures in fetuses with left CDH without significant congenital heart disease, from a large group of fetuses undergoing comprehensive evaluation; our proposed normative curves are left-shifted compared to standardized curves from unaffected fetuses (29). And, although left heart development is substantially impacted, the degree of fetal left heart underdevelopment alone was not related to survival in this single center study. Use of these CDH-specific normative values when assessing a fetus with CDH should help the clinician to better interpret the echocardiographic data present in the fetal period and to avoid erroneously arriving at a diagnosis of structural heart disease when in fact the heart is normal for this condition.

The lack of association between left heart size and survival in this contemporary cohort is consistent with prior reports (5, 23). Z-score differences of up to 0.3 ± 1.0 would require 190–200 fetuses to demonstrate a statistically significant difference between survivors and non-survivors if ∼2/3 of the fetuses survive after birth, but even a proposed cut-off z-score <−1 provided only modest outcome prediction. Of left heart structures, Vogel and colleagues found only AoV diameter, with a cut-off z-score <−2 (curves from unaffected fetuses), was associated with survival, although it was not an independent predictor (23). Multiple investigators have hypothesized that distorted anatomy and compression due to herniated thoracic contents account for differences in left heart size and output, primarily supported by the observations by ourselves and others that alterations in fetal blood flow patterns are related to fetal anatomic measures of CDH severity (15, 16, 23–25). In support of this decrease in left heart output being physiologically important, we have recently reported that middle cerebral artery (MCA) pulsatility index values are significantly lower in fetuses with L-CDH compared to normal control fetuses, and that lower left heart output was correlated with lower MCA vascular impedance (24). The neurodevelopmental effect of such changes in MCA-PI in response to decreased LVCO is unknown.

A growing body of literature is focused on development of the pulmonary circulation and fetal pulmonary vascular hypoplasia, aimed to better understand pathophysiology and provide more precise information for decision-making (4–8, 21–25, 30). Fetal blood flow alterations in CDH may be related to pulmonary vascular physiology in addition to intracardiac fetal blood flow and compressive effects. Fetal lung hypoplasia is associated with structural and functional pulmonary vascular changes, including alterations in hemodynamic parameters and reactivity (4, 7, 31–33). Endothelial nitric oxide synthase expression is decreased in fetal hypoplastic lungs, consistent with demonstrated lack of response to oxygen (mediated *via* nitric oxide) (4, 8, 33, 34). Recently, we demonstrated additional alterations in the fetal environment in CDH, with differences in levels of cord blood growth factors and inflammatory mediators, related to the persistence of neonatal PH, also associated with anatomic markers of severe CDH (35, 36). Thus, fetal blood flow alterations and abnormal vascular reactivity in CDH could mimic the circulatory derangements of PPHN due to increased pulmonary vascular resistance (PVR); relatively increased fetal PVR from a small pulmonary vascular bed and vascular biochemical changes will result in decreased left-sided blood flow and ventricular loading (16). The neonatal increase in left-sided structures following CDH repair may be attributable to normalization of both mechanical and hemodynamic mechanisms, as PVR decreases over time (23, 36, 37). These hemodynamic effects are consistent with the paradigm wherein blood flow contributes to fetal cardiac development (22, 23, 38).

Interestingly, we demonstrated poorer left heart development with advancing gestation by survival status, consistent with prior fetal cardiac studies (19), that showed more pronounced abnormality at later gestation and as was previously shown for other fetal CDH anatomic markers that portend worse prognosis (5, 6, 39, 40). LV width was the most impacted left heart measurement in our cohort. In addition to the abnormalities in non-survivors, the CDH-specific LV width distribution demonstrated the greatest difference of all of our measurements from the distribution derived from unaffected fetuses. It may be that LV width, reflecting LV filling, is impacted by both anatomic compression and fetal pulmonary vascular physiology, whereas other measures may be more affected by a single mechanism. We speculate that the relationship between anatomic CDH severity and left heart development results in unfavorable cardiopulmonary interactions at birth, but fetal ultrasound measurements may be too crude to identify the distinct contribution of these factors.

Consistent with the physiology of fetal intervention for CDH (accelerated fetal lung growth), tracheal occlusion can impact fetal markers of severity (8, 41). Left heart structures relatively increase in size (with increased preload) and, pulmonary vascular responsiveness is restored among survivors (8, 27). Fetal tracheal occlusion also improves some neonatal outcomes, including survival and decreased incidence of PPHN, supporting the relationship of left heart development to pulmonary vascular physiology (42, 43).

### Limitations

Our findings are based on single-center data, which may limit generalizability. However, experienced ultrasonographers may be required for the consistent ascertainment of important fetal markers of CDH severity, with strong agreement between expert evaluators (5, 8, 27, 44–47). Our prior work demonstrated acceptable interobserver variability for the measurements in the current study (25, 44). Further, our data are confined to left CDH (more common than right CDH), consistent with prior fetal studies. However, evaluation of data from fetuses with right CDH could be useful, as we previously demonstrated fetal hemodynamic alterations are more pronounced in left CDH, compared to right CDH (25). Given the smaller numbers of these fetuses evaluated at any single center, collaborations among experienced centers may be informative. Finally, the number of fetuses that ultimately were used to generate our normative curves was not large, with observations obtained predominantly during mid-gestation. However, this is the usual timing of the mid-gestation routine anatomic ultrasound evaluation that identifies the CDH and thus the most common time that these fetuses are referred for clinical evaluation, and our curves demonstrate similar shape to those generated from unaffected fetuses.

## Conclusion

Normative data on left heart structures from fetuses with left CDH without congenital heart disease result in z-scores that are significantly left-shifted compared to normal curves from unaffected fetuses. We suggest the use of these CDH-specific equations during fetal evaluation, as this may mitigate antenatal concern regarding structural left heart disease, since measurements of these structures in CDH have been shown to normalize after birth (23). However, the development of left heart structures in fetuses with CDH and without congenital heart disease has limited bearing on survival after birth, as an isolated consideration. Future work could evaluate the relationship of these measurements to other neonatal outcomes, including cardiac physiology and diastology (representing ventricular interaction) (17, 48), and the persistence of pulmonary hypertension, as well as the combination of left heart markers with additional fetal anatomic and physiologic measurements, to refine fetal risk for adverse neonatal outcomes.

## Data Availability

The raw data supporting the conclusions of this article will be made available by the authors, without undue reservation.
