# Supplementary material for: Expected small left heart size in the presence of congenital diaphragmatic hernia: Fetal values and Z-scores for infants confirmed to have no heart disease postnatally
Source: Front Pediatr. 2022 Dec 6;10:1083370. doi: 10.3389/fped.2022.1083370 (PMC9763578; doi:10.3389/fped.2022.1083370)

Supplement

Keller R et al: Left heart size in the presence of congenital diaphragmatic hernia: normal fetal values

Table S1. Equations for prediction of echocardiographic parameters in fetuses with left CDH for all fetuses (survivors and non-survivors).

**Parameter**	**Coefficient (β)***	**Constant**	**Root MSE**	**Adjusted R^2**
MV diameter (n=142)	1.1406	-4.2304	0.1677	0.50
AV diameter (n=133)	1.1036	-4.6657	0.1532	0.52
LV length (n=140)	1.1251	-3.2114	0.1743	0.47
LV width (n=124)	1.1221	-4.7550	0.2935	0.24
TV diameter (n=142)	1.1798	-4.0820	0.1702	0.51
PV diameter (n=136)	1.2028	-4.5913	0.1243	0.66

* All coefficients were statistically significant ($p<0.001$).
Mean predicted value = exp [(β * gestational age) + constant]
MV mitral valve; AV aortic valve; LV left ventricle; TV, tricuspid valve; PV, pulmonic valve

Figure S1. Left heart parameters (data derived from all fetuses, survivors and non-survivors): distribution of z-scores and kernel density estimates from fetuses with left CDH derived from 1) published data from unaffected fetuses[29] (white bars, solid line) and 2) left CDH-specific data (gray bars, dashed line). CDH-specific equations were derived with data from all fetuses, regardless of survival after birth, ***a***, mitral valve diameter, n=142; ***b***, aortic valve diameter, n=133; ***c***, left ventricle length, n=140; ***d***, left ventricle width, n=124.

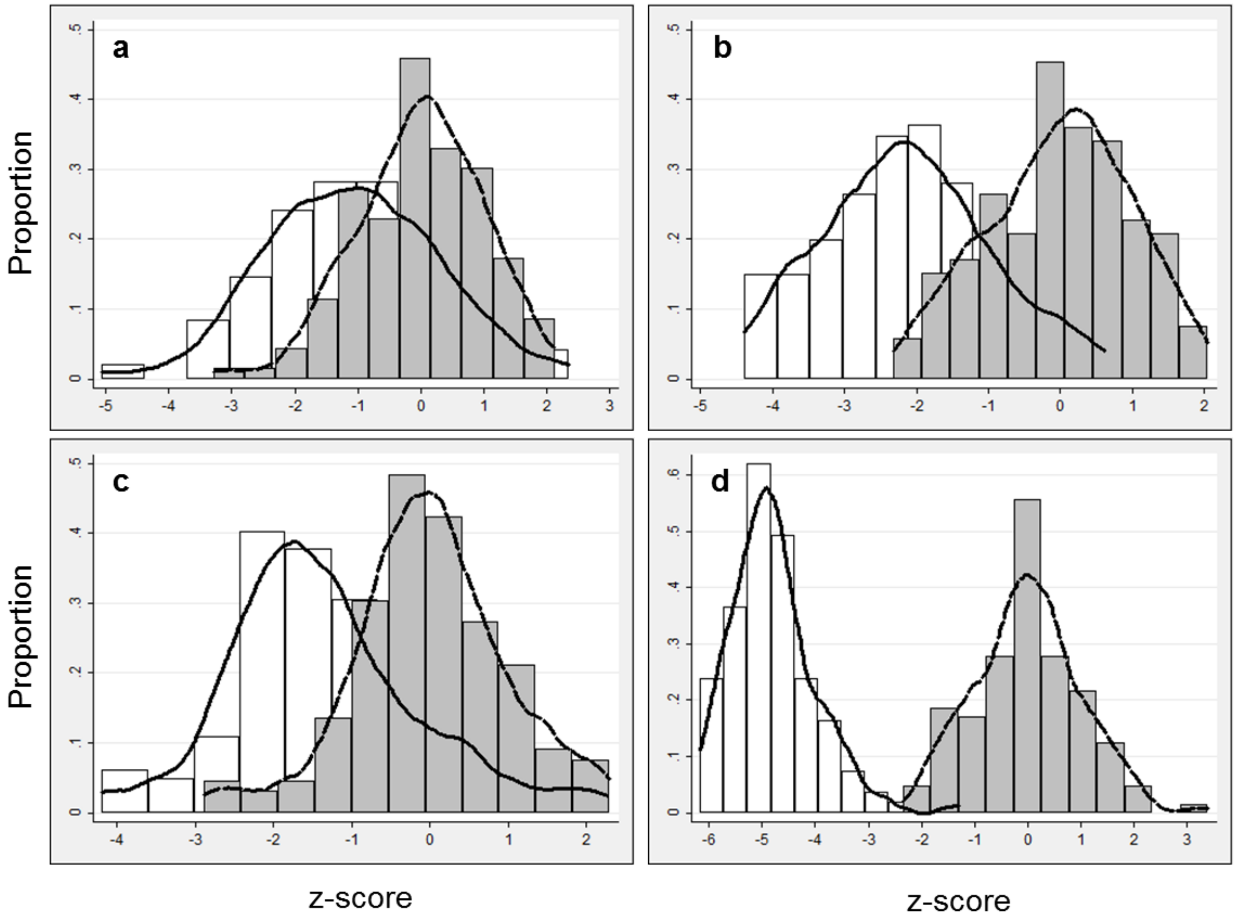

Supplement: Supplementary file 1 [file Datasheet1.pdf]
